# Arsenic removal performance and mechanism from water on iron hydroxide nanopetalines

**DOI:** 10.1038/s41598-022-21707-1

**Published:** 2022-10-14

**Authors:** Yulong Wang, Lin Zhang, Chen Guo, Yali Gao, Shanshan Pan, Yanhong Liu, Xuhui Li, Yangyang Wang

**Affiliations:** 1 National Demonstration Center for Environmental and Planning, College of Geography and Environmental Science, Henan University, Kaifeng, 475004 China; 2 Key Laboratory of Geospatial Technology for the Middle and Lower Yellow River Regions (Henan University), Ministry of Education, Kaifeng, 475004 China; 3 Henan Engineering Research Center for Control and Remediation of Soil Heavy Metal Pollution, Henan University, Kaifeng, 475004 China; 4 College of Software, Henan University, Kaifeng, 475004 China

**Keywords:** Pollution remediation, Chemical engineering

## Abstract

Human health has been seriously endangered by arsenic pollution in drinking water. In this paper, iron hydroxide nanopetalines were synthesized through a precipitation method using KBH_4_ and their performance and mechanism of As(V) and As(III) removal were investigated. The prepared material was characterized by SEM–EDX, XRD, BET, zeta potential and FTIR analyses. Batch experiments indicated that the iron hydroxide nanopetalines exhibited more excellent performance for As(V) and As(III) removal than ferrihydrite. The adsorption processes were very fast in the first stage, followed a relatively slower adsorption rate and reached equilibria after 24 h, and the reaction could be fitted best by the pseudo-second order model, followed by the Elovich model. The adsorption isotherm data followed to the Freundlich model, and the maximal adsorption capacities of As(V) and As(III) calculated by the Langmuir model were 217.76 and 91.74 mg/g at pH 4.0, respectively, whereas these values were 187.84 and 147.06 mg/g at pH 8.0, respectively. Thermodynamic studies indicated that the adsorption process was endothermic and spontaneous. The removal efficiencies of As(V) and As(III) were significantly affected by the solution pH and presence of PO_4_^3–^ and citrate. The reusability experiments showed that more than 67% of the removal efficiency of As(V) could be easily recovered after four cycles. The SEM and XRD analyses indicated that the surface morphology and crystal structure before and after arsenic removal were stable. Based on the analyses of FTIR, XRD and XPS, the predominant adsorption mechanism was the formation of inner-sphere surface complexes by the surface hydroxyl exchange reactions of Fe–OH groups with arsenic species. This research provides a new strategy for the development of arsenic immobilization materials and the results confirm that iron hydroxide nanopetalines could be considered as a promising material for removing arsenic from As-contaminated water for their highly efficient performance and stability.

## Introduction

Arsenic (As) is a naturally ubiquitous carcinogenic metalloid in the environment, including the atmosphere, sediments, soil, minerals, groundwater, and food^1^. It primarily occurs in the forms of two oxidation states, arsenate (As(V)) and arsenite (As(III)). The release of As originating from geogenic sources and anthropogenic activities to water can result in the elevated As groundwater used for drinking, which endangers ecosystem security and human health for the accumulation through the food chain^2–4^. In light of its high toxicity and accumulation, the World Health Organization proposed in 1993 that the maximum contaminant limit of arsenic for drinking (WHO-MCL) cannot be over 10 μg/L^5^. Up to now, over 200 million people are at risk of consuming arsenic-contaminated groundwater, whereas in China, approximately 19.6 million people are affected by this risk^6,7^. Therefore, it is an urgent requirement to develop water treatment technologies that meet the requirements of drinking water.

Over the past decades, numerous purification techniques, including ion-exchange, biological treatment, coagulation-precipitation, adsorption and membrane filtration, have been utilized for arsenic removal from contaminated waters^8–14^. Among these techniques, adsorption is always considered as the most promising and economical method because of its easy operation, low-cost and high treatment efficiency^15^. Actually, great explorations have resulted in the development of a large number of adsorbents with improved removal efficiencies and adsorption capacities for arsenic removal. Metal oxides including iron hydroxides, polyalumina, manganese oxides, zirconium oxides, lanthanum hydr(oxide)s and cerium oxides are attractive for their strong affinity and high capacities for arsenic species^16–24^. Iron oxides, for example, ferrihydrite, goethite, akaganéite, magnetite, hematite, etc., are the most abundant metal oxides on the surface of the earth environments and the adsorption of arsenic onto iron oxides has aroused widespread interest for their use in the decontamination of arsenic owing to several additional advantages, such as their environmental friendliness, lower cost and higher natural abundance^25–27^. However, these iron oxides possess tremendous discrepancies in arsenic removal capacities and efficiencies, which could be attributed to their varieties of crystallinity and numbers of surface adsorption sites^20^.

Poorly crystalline Fe oxyhydroxide nanoparticles with large specific surface areas, known as ferrihydrite, are widely occurring in natural environments, and exhibited higher arsenic uptake capacity than goethite, magnetite and hematite^20,27^. The excellent adsorption capacity of arsenic by ferrihydrite could be due to the nanoscale of its particles, poorly crystalline and high specific surface area^20,28,29^. The adsorption behaviour and mechanisms of As(V) and As(III) on ferrihydrite have been widely performed with the help of batch adsorption experiments and spectroscopic techniques^20,21,28–31^. Many environmental factors, such as solution pH, background electrolyte concentration, adsorption temperature, competing cations/anions and different precipitated reaction media, have been also considered^29,31–33^. These results indicated that inner-sphere surface complexes formed by ligand exchange reactions were the predominant adsorption mechanisms of As(V), while As(III) forms both inner-sphere and outer-sphere surface complexes^28,30^. As(V) and As(III) adsorption were pH-dependent: the adsorption amounts of As(V) decreased with the increases of solution pH, while those of As(III) exhibited the maximum adsorption capacity at pH 7‒9^20,29^. What’s more, the pore structure of ferrihydrite aggregates could be changed by the processes of freezing and thawing, which can further influence cation and anion adsorption onto ferrihydrite aggregates^32,33^. However, to our best knowledge, no studies have focused on the corresponding relation between removal efficiencies and surface morphology of ferrihydrite. Furthermore, the agglomeration of ferrihydrite is the major problem, which reduces its contaminant (such as arsenic) removal efficiencies, and subsequently, limits its application^34^. Recently, synthesis of nanomaterials on a suitable support medium is considered to be an effective way^35–37^. Nevertheless, these composite materials have lower adsorption capacities of arsenic. For example, the maximum adsorption capacity by the starch functionalized maghemite nano-adsorbents was 8.57 mg/g^35^, the maximum adsorption capacity of iron oxide coated hollow poly(methylmethacrylate) microspheres was 10.031 mg/g^36^, and the biochar composite impregnated with Zn and Al oxides exhibited the maximum As(III) and As(V) adsorption capacities of 10.728 and 11.786 mg/g, respectively^37^. The lower As adsorption capacities restrict the treatment of arsenic-containing water, especially the high concentrations of arsenic. What’s more, generous As-containing solid waste generated by the arsenic removal using these composite materials is also difficult to manage. Consequently, further improved approaches, such as the prepared method of ferrihydrite, are required to explore to reduce the agglomeration of ferrihydrite and improve its arsenic removal efficiencies.

In this paper, we proposed a preparation method of iron hydroxide nanopetalines through a precipitation method using KBH_4_. The iron hydroxide nanopetalines were characterized by scanning electron microscopy (SEM) with energy-dispersive X-ray spectrometer (EDX), Brunauer–Emmett–Teller (BET) surface area analysis and X-ray diffraction (XRD) analyses indicated that the main phase of the iron hydroxide nanopetalines was ferrihydrite. The removal performance and mechanism of As(V) and As(III) were subsequently studied using batch adsorption experiments, Fourier transform infrared spectroscopy (FTIR), XRD and X-ray photoelectron spectra (XPS) analyses. This research provides a new synthesis strategy of materials and commercial potential application of poorly crystalline Fe hydroxides.

## Materials and methods

### Materials

All chemicals employed in this study were analytical grade and used without further purification. Fe(NO_3_)_3_∙9H_2_O, KBH_4_ and NaOH were purchased from Sinopharm Chemical Reagent Co. (Shanghai, China). Deionized (DI) water was used to prepare working solutions for all experiments. All glass containers were first cleaned by soaking in a 5% HNO_3_ solution for at least 24 h and rinsed several times with DI water prior to the experiments. The stock solutions of As(V) and As(III) at a concentration of 1.0 g/L were obtained by dissolving As_2_O_5_ and As_2_O_3_ into DI water, respectively, and were diluted to obtain arsenic working solutions with the required concentrations using DI water.

### Preparation of the adsorbent

The iron hydroxide nanopetalines were synthesized via a precipitation process using KBH_4_. Briefly, 8.08 g (0.02 mol) of Fe(NO_3_)_3_·9H_2_O was dissolved in 40 mL of DI water under magnetic stirring to obtain a 0.5 M Fe^3+^ solution. Then, 4.32 g (0.08 mol) of KBH_4_ was dissolved in 80 mL of DI water, and the solution was then added in a dropwise manner into the abovementioned Fe^3+^ solution. After the addition, the resulting suspension was continuously stirred for at least 3 h. Finally, the suspension was rinsed with DI water followed by anhydrous ethanol. The obtained precipitate was dried in a vacuum oven at 50 °C for 24 h. The dried product was crushed and stored in a desiccator for further analyses.

### Characterization of adsorbents

The morphologies and surface elemental compositions of the adsorbent before and after adsorption were inspected by a field emission scanning electron microscopy with energy dispersive X-ray spectroscopy (SEM–EDX, Hitachi S-4800, Japan). Powder X-ray diffraction (XRD) patterns of the solid samples were taken on a D8 Advance X-ray diffractometer (Bruker-AXS, Germany) using Cu Kα radiation (λ = 1.5406 Å) at 40 mA and 40 kV with 2*θ* increments of 0.05°.

The specific surface areas and pore structures of the materials were analyzed by N_2_ adsorption/desorption using a Micromeritics ASAP 2460 surface area analyzer (Micromeritics ASAP 2460, USA) at 77 K and were calculated by the Brunauer–Emmett–Teller (BET) method with the adsorption curves and Barrett–Joyner–Halenda (BJH) model with the desorption branches, respectively. FTIR spectra were carried out using a Fourier transform infrared spectrometer with a transmission model (Thermo Nicolet 6700, USA) using the KBr pellet method. X-ray photoelectron spectra (XPS) data were determined by an X-ray photoelectron spectra spectrometer (Thermo ESCALAB 250Xi, USA). The spectra were acquired by a monochromatized Al K*α* X-ray source (*hν* = 1486.6 eV) and a hemispherical electron analyzer and the binding energy was calibrated based on the C 1s photoelectron peak (284.6 eV). A zeta potential analyzer (Nano Zetasizer 2000, Malvern Co., UK) was employed to analysis the pH of the point of zero charge (pH_pzc_) of the adsorbent, as determined by measuring the ζ-potential.

### Batch adsorption experiments

Arsenic removal experiments were performed by the batch equilibrium method at room temperature (25 ± 1 °C) with various initial arsenic concentrations and an adsorbent dose of 0.5 g/L. Adsorption isotherms, adsorption kinetics and the effect of pH and competitive anions were conducted to evaluate the removal performance of As(V) and As(III) species by the adsorbent. The solution pH was fixed at required values throughout the experiments by addition of NaOH or HCl solution. After in a thermostatic shaker at 180 rpm for 24 h, the suspensions were filtered through 0.22 μm membrane syringe filters to determine the residual arsenic concentrations in solution. The concentrations of arsenic in aqueous solution were determined by a hydride-generation atomic fluorescence spectrophotometer (AFS-3100, Haiguang Corp., Beijing). All experiments were carried out in triplicate, and the average values were reported to represent the results. The As adsorption capacity removed by the adsorbent was determined as follows:

1 $$q_{{\text{e}}} = \frac{{(C_{0} - C_{e} ) \times V}}{m}$$

where *C*_0_ (mg/L) and *C*_e_ (mg/L) are the initial and equilibrium concentrations of As, respectively; *V* is the volume of the As solution (L); and *m* is the mass of the adsorbent (g).

The influence of solution pH on As(V) and As(III) removal was carried out in the initial As concentrations of 75 mg/L with pH values ranging from 3 to 11. The adsorption kinetics studies were measured by adding 0.125 g of adsorbent into a 250 mL As solution with initial concentrations of 75 mg/L. Approximately 2 mL aliquots of suspension were sampled at different time intervals. The adsorption isotherms were examined at pH 4.0 and 8.0 with different initial concentrations of As(V) and As(III) solution (30.0‒140.0 mg/L). To evaluate the effect of common interfering anions, SO_4_^2−^, CO_3_^2−^, PO_4_^3−^ and citrate ions used as competitive ions were respectively added to 75 mg/L As solutions at pH 7.0. In the reusability study, adsorption–desorption experiments were conducted for four cycles at pH 7.0 with initial As concentrations of 75 mg/L, and the desorption experiments were tested using 2.0 mol/L NaOH solution over 4 h.

## Results and discussion

### Characterization of materials

#### Scanning electron microscopy

**Figure 1:**
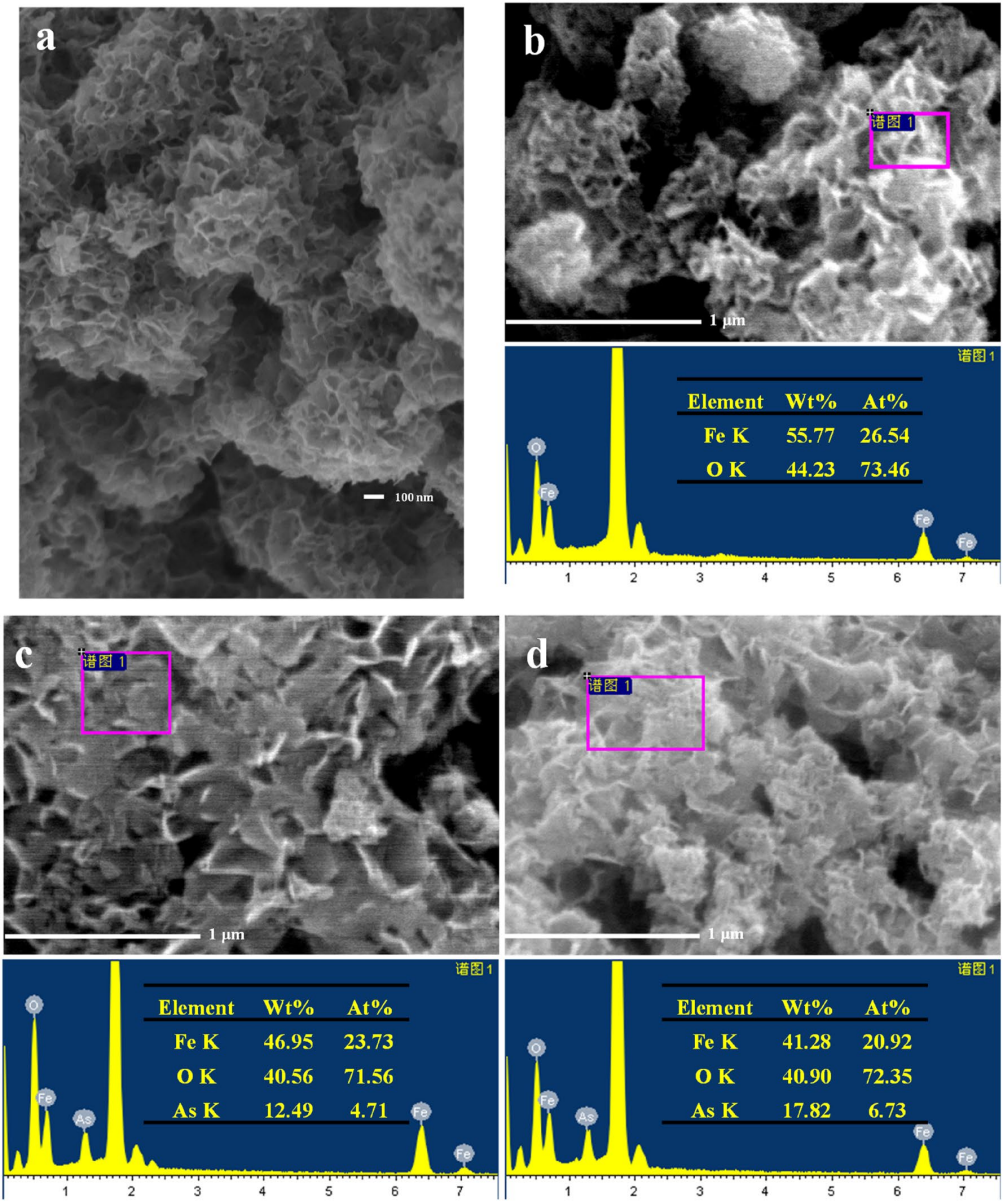
(**a**) SEM micrograph of the iron hydroxide nanopetalines. EDX analyses of the iron hydroxide nanopetalines (**b**) and those after As(III) (**c**) and As(V) (**d**) adsorption. Initial As concentration was 75.0 mg/L, solution pH was 4.0, and adsorbent dose was 0.5 g/L.

The particle surface morphology of the iron hydroxide nanopetalines were performed by SEM. As presented in Fig. 1a, the SEM image shows that the particles of the sample were aggregated by petaline-like particles. Due to the aggregation of the iron hydroxide nanopetalines, heterogeneously and irregularly distributed micropore channels with different sizes were formed on the surface of the particles. Figure 1b–d illustrate the elemental distributions of the virgin iron hydroxides and those after As(V) and As(III) adsorption at pH 4.0 with the initial As concentrations of 75 mg/L, respectively. As shown in Fig. 1b–d, no significant changes have been observed on the surface of particles after arsenic removal, which indicated that the physical structures of the material were stable and that the iron hydroxide nanopetalines were suitable for the treatment of arsenic removal from water. Based on the EDX analysis, only iron and oxygen elements were detected on the surface of the original adsorbent, and their contents were 26.54 and 73.46 At%, respectively. Although KBH_4_ was used to prepare the material, boron element was not checked out by SEM–EDX, which could be attributed to boron being below the detection limit. Furthermore, the content of boron was also determined by inductively coupled plasma optical emission spectrometry (ICP-OES, iCAP-6000, America) after digestion with dilute nitric acid (1 mol/L HNO_3_), resulting in a value of 15 mg/g. After the uptake of arsenic, the Fe concentrations on the surface were decreased from 26.54 to 23.73 and 20.92 at% and the As concentrations were approximately 4.71 and 6.73 at% for As(III) and As(V), respectively. This result indicated that the adsorption capacity of As(V) by the adsorbent was much higher than that of As(III), which was further confirmed by the results of batch adsorption experiments, as shown below in Figs. 3 and 4.

#### X-ray diffraction

**Figure 2:**
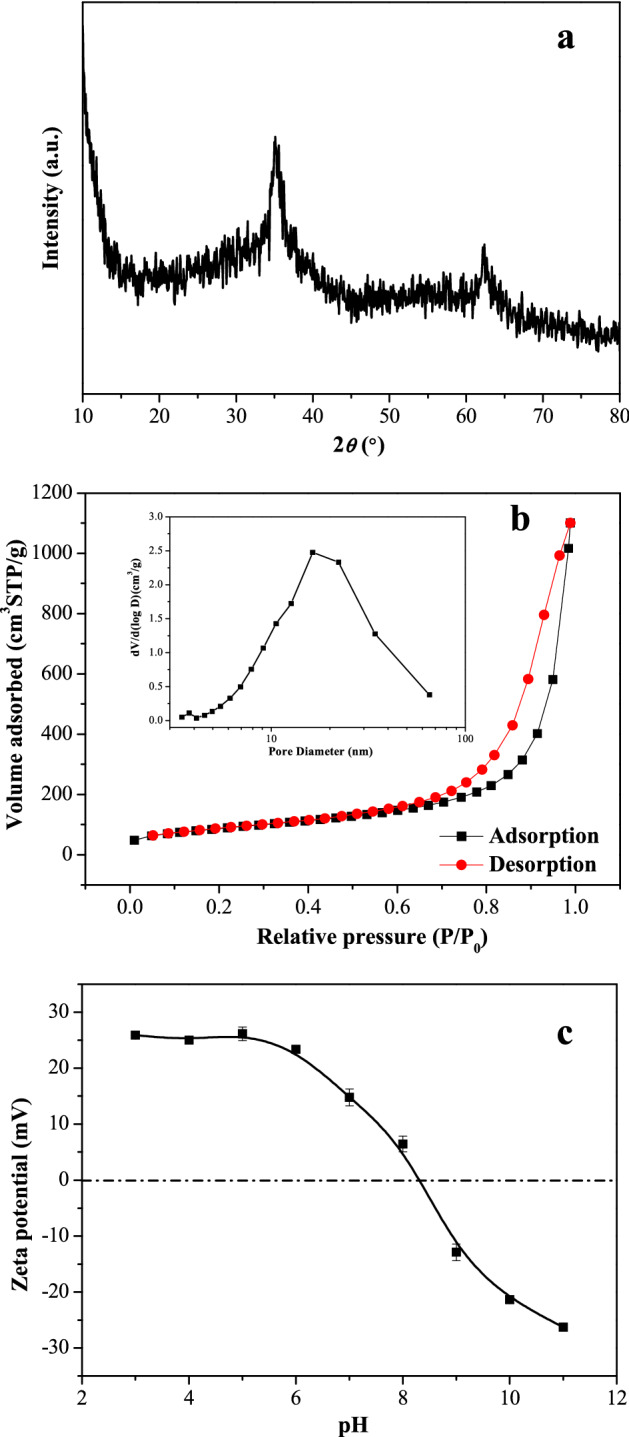
XRD pattern (**a**), N_2_ adsorption–desorption isotherms and pore size distributions (inset) based on BJH analysis (**b**) and zeta potentials (**c**) as a function of pH of the iron hydroxide nanopetalines.

The crystallinity of the material was characterized by XRD analysis as depicted by Fig. 2a. The spectrum shows two broad bands at approximately 35.2° and 62.4°, respectively, which could be attributed to the characteristics of poorly ordered two-line ferrihydrite, as suggested by its name^21,38,39^. These observations indicated that the predominant phase of iron hydroxide nanopetalines was poorly crystallized ferrihydrite.

#### Specific surface area

The N_2_ adsorption–desorption isotherms and the pore size distributions determined by the BJH analysis using the desorption data of the adsorbent are given in Fig. 2b. As depicted in Fig. 2b, the adsorption–desorption isotherms of the adsorbent did not exhibit a plateau at high p/p_0_ values. According to the IUPAC classification, its isotherms could be assigned to type IV isotherms with H3-type hysteresis loops, indicating the adsorption of N_2_ by these pores, which were produced by aggregation of the platelet-like particles, was multilayered physical adsorption^38,40^. The adsorption isotherms of the adsorbent did not exhibit a plateau at high p/p_0_ values. These results revealed that the material was mesoporous, as illustrated in the inset in Fig. 2b. The specific surface areas and *t*-plot external micropore areas calculated by the BET method were 317.07 and 321.96 m^2^/g, respectively, and the BJH model was employed to determine the pore size distribution and total pore volume based on the desorption branch. The results showed that the average pore diameter and total pore volume were 11.42 nm and 1.706 cm^3^/g, respectively. These parameters obtained from the N_2_ adsorption–desorption isotherms are summarized in Table 1. A previous study indicated that the specific surface area of ferrihydrite aggregates formed by freezing and thawing was between 320 and 380 m^2^/g^32^.

**Table 1:** BET specific surface area and porosity measurements of the material.

Adsorbent	Specific surface area (m^2^/g)	*t*-plot micropore area (m^2^/g)	Average pore diameter (nm)	Average pore volume (cm^3^/g)
Iron hydroxide anopetalines	317.07	321.96	11.42	1.706

#### Point of zero charge

Figure 2c shows the surface zeta potentials of the iron hydroxide nanopetalines at different pH values. It can be obviously observed that the pH_pzc_ value of the material was found to be approximately 8.28. This result was consistent with that of ferrihydrite reported in previous literatures^38,41^. As presented in Fig. 2c, the solution pH could dramatically influence the surface charge of the material. When the solution pH was above its pH_pzc_, the surface of the adsorbent was negatively charged, resulting into a stronger electrostatic repulsion between arsenic and the active sites of the material. This would not be conducive to arsenic removal. On the other hand, the surface charge became positive at pH < pH_pzc_, and electrostatic attraction between arsenic and the active sites was enhanced, which would be advantageous to arsenic adsorption onto the adsorbent.

### Batch adsorption experiments

#### Adsorption kinetics

**Figure 3:**
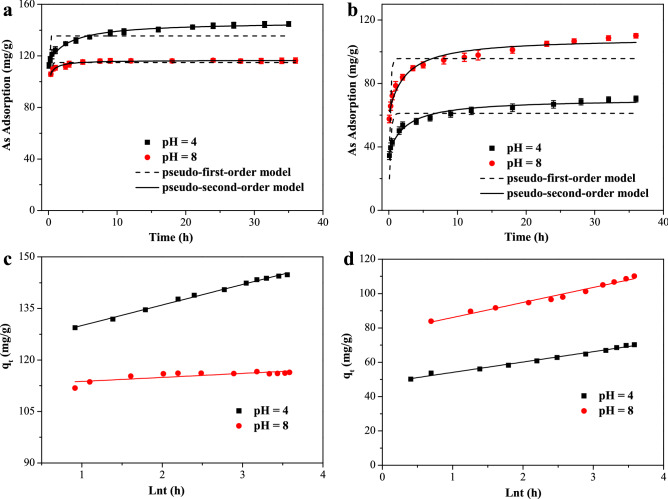
Adsorption kinetics of As(V) (**a**) and As(III) (**b**) adsorption and kinetics plot of pseudo-first-order model and pseudo-second-order model at pH 4.0 and 8.0 with initial As concentrations of 75 mg/L and dosage of 0.5 g/L. Elovich model of adsorption kinetics of As(V) (**c**) and As(III) (**d**).

As illustrated in Fig. 3a,b, the adsorption kinetics of As(V) and As(III) onto iron hydroxide nanopetalines at pH 4.0 and 8.0 with initial As concentrations at 75 mg/L were performed in order to assess the arsenic adsorption rate. It is obvious that the initial adsorption process was extremely fast, and more than 80% of the equilibrium adsorption capacity was achieved in the first 6 h. After that, the adsorption rate gradually slowed down and equilibria were reached after 24 h. The adsorption processes were rapid at the preliminary stage because of the large number of unoccupied adsorption sites, which was advantageous for the transport of arsenic onto the surface of the iron hydroxides^42^. The decrease of vacant sites resulted in the subsequent slower phase, in which intraparticle diffusion and surface precipitation were the predominant mechanisms for the uptake of arsenic^12,31^.

Pseudo-first-order, pseudo-second-order and Elovich models were used to fit the adsorption kinetics data in order to investigate the characteristics of the adsorption process. The linear forms of these models can be described as follows^40,42^:

2 $$\ln (q_{e} - q_{t} ) = \ln q_{e} - k_{1} t$$

3 $$\frac{t}{{q_{{\text{t}}} }} = \frac{1}{{k_{2} q_{{\text{e}}}^{2} }} + \frac{t}{{q_{{\text{e}}} }}$$

4 $$q_{{\text{t}}} = \frac{1}{\beta }\ln (\alpha \beta ) + \frac{1}{\beta }\ln t$$

where *q*_e_ (mg/g) and *q*_t_ (mg/g) are the adsorption amounts of arsenic at equilibrium and at time *t*, respectively, and *k*_1_ (h^−1^) and *k*_2_ (g/(mg∙h)) are the rate constants of the pseudo-first-order and pseudo-second-order models, respectively. *α* (mg/g/h) and *β* (g/mg/h) are the Elovich coefficients, which are the initial adsorption rate and the desorption constant related to the extent of surface coverage and the activation energy of chemisorption, respectively^35,43^. The fit quality was estimated by the determination coefficient (*R*^2^) and the average relative error (ARE)^44^. Table 2 represents the fitting parameters and correlation coefficients, as well as the values of the ARE. The results indicated that the kinetics data was best simulated by the pseudo-second order model, followed by the Elovich model (Fig. 3c,d). Compared to the pseudo-first-order model, the pseudo-second-order model described the arsenic adsorption processes better, as indicated by the higher correlation coefficient (*R*^2^ > 0.96). This suggested that the dominant adsorption processes of arsenic were controlled by the chemisorption process^10,42^. At pH 4.0, the calculated *k*_2_ value of As(V) adsorption was higher than that of As(III) adsorption, indicating that the removal of As(V) was faster than that of As(III) at pH 4.0. While at pH 8.0, the nearby *k*_2_ values of As(V) and As(III) adsorption suggested that the rates of As(V) and As(III) removal were close. These results indicated that the solution pH was an important factor affecting the adsorption performance, which was further studied below (Fig. 5a). Furthermore, the better fit by the Elovich model suggested that the heterogeneous adsorption occurred on the surface of the material^45^.

**Table 2:** Adsorption kinetics parameters for As(V) and As(III) adsorption on iron hydroxide nanopetalines at pH 4.0 and 8.0.

Models	As(V)		As(III)	
	pH 4.0	pH 8.0	pH 4.0	pH 8.0
**Pseudo-first-order model**				
*k*_1_ (h^−1^)	20.43	7.36	4.71	6.10
*q*_e_ (mg/g)	135.51	114.91	61.25	95.67
*R*^2^	0.769	0.842	0.876	0.843
ARE (%)	15.65	3.78	3.63	3.35
**Pseudo-second-order model**				
*k*_2_ (g/(mg h))	0.0259	0.0136	0.0058	0.0126
*q*_e_ (mg/g)	146.41	116.69	70.32	108.81
*R*^2^	0.984	0.961	0.973	0.961
ARE (%)	1.65	1.13	1.87	1.31
**Elovich model**				
*α* (mg/g/h)	6.14 × 10^9^	7.18 × 10^41^	1.74 × 10^4^	6.01 × 10^4^
*β* (g/mg/h)	0.167	0.855	0.166	0.114
*R*^2^	0.965	0.607	0.958	0.957
ARE (%)	0.201	1.02	0.938	1.73

#### Adsorption isotherms

**Figure 4:**
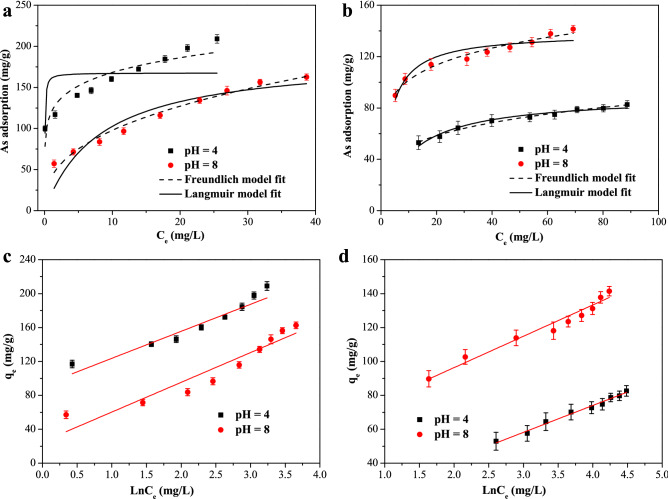
Adsorption isotherms of As(V) (**a**) and As(III) (**b**) adsorption at pH 4.0 and 8.0 with initial As concentrations ranging from 30 to 140 mg/L and dosage of 0.5 g/L. Temkin adsorption isotherm models of As(V) (**c**) and As(III) (**d**).

Adsorption isotherms were employed to evaluate the maximum adsorption capacities of As(V) and As(III) on iron hydroxide nanopetalines. The influences of initial As concentrations ranging from 30 to 140 mg∙L^−1^ on As(V) and As(III) removal were carried out at pH 4.0 and 8.0, and the corresponding results are illustrated in Fig. 4a,b. It can be seen that the adsorption capacities increased as the initial As concentrations increased. The Langmuir, Freundlich and Temkin adsorption isotherm models, were applied to simulate the adsorption isotherm data. The linear expressions of these isotherm models could be represented in the following^40,42^:

5 $$\frac{{C_{e} }}{{q_{e} }} = \frac{1}{{q_{\max } }}C_{e} + \frac{1}{{K_{{\text{L}}} q_{\max } }}$$

6 $$\log q_{e} = \log K_{{\text{F}}} + \frac{1}{n}\log C_{{\text{e}}}$$

7 $$q_{e} = \frac{RT}{b}\ln A + \frac{RT}{b}\ln C_{{\text{e}}}$$

where *C*_e_ (mg/L) is the arsenic concentration at equilibrium, *q*_e_ (mg/g) and *q*_max_ (mg/g) stand for the equilibrium and maximum adsorption capacities, respectively, *K*_L_ (L/mg) is the Langmuir model constant, *K*_F_ ((mg/g)·(mg/L)^–1/*n*^) and *n* are the adsorption constants of the Freundlich model. *K*_L_ and *K*_F_ roughly represent the adsorption affinity between arsenic and the adsorption sites. 1/*n* is a heterogeneity factor representing the adsorption intensity. *A* (L/g) and *b* (J/mol) are the Temkin binding constants related to the enthalpy of adsorption, *R* and *T* (*K*) are the Universal Gas constant and the absolute temperature, respectively.

The fitting curves of the experimental data are also displayed in Fig. 4a‒d and the calculated isotherm parameters are represented in Table 3. As illustrated in Table 3, the higher regression coefficients (*R*^2^ > 0.99) and the lowest ARE (< 6%) suggested that the Freundlich model fitted the experimental data best. This indicated that the surface of the adsorbent likely had heterogeneous adsorption sites and that the adsorption energies of arsenic adsorption on the surface of the adsorbent were different^17^. This result was different from the fitting result of arsenic adsorption on pure ferrihydrite according to the report by Jiang et al.^21^. The adsorption isotherms of pure ferrihydrite were described better by the Langmuir isotherm model than by the Freundlich model. This discrepancy between our iron hydroxide nanopetalines and pure ferrihydrite could be due to the use of KBH_4_ in the synthesis process, which led to a more heterogeneous surface. The calculated Langmuir adsorption capacities for As(V) at pH 4.0 and 8.0 were 217.76 and 187.84 mg/g, respectively, and those for As(III) at pH 4.0 and 8.0 were 91.74 and 147.06 mg/g, respectively. These results were in consistent with the results of the adsorption kinetics study (Fig. 3a,b). For the simulation of the Temkin model, the values of bonding energy (b) ranged from 70.61 to 155.82 J/mol, suggesting that the arsenic removal process was both chemisorption and physisorption^40,45^.

**Table 3:** Adsorption isotherm parameters of As(V) and As(III) on iron hydroxide nanopetalines at pH 4.0 and 8.0 with initial As concentrations ranging from 30 to 140 mg/L.

Models	As(V)		As(III)	
	pH 4.0	pH 8.0	pH 4.0	pH 8.0
**Langmuir model**				
*q*_max_ (mg/g)	217.76	187.84	91.74	147.06
*k*_L_ (L/mg)	0.500	0.124	0.0837	0.196
*R*^2^	0.881	0.965	0.989	0.974
ARE (%)	14.37	12.34	10.59	12.66
**Freundlich model**				
*k*_F_ ((mg/g)·(mg/L)^–1/*n*^)	120.03	40.81	29.21	70.63
1/*n*	0.146	0.379	0.231	0.159
*R*^2^	0.994	0.992	0.993	0.997
ARE (%)	5.58	4.97	5.13	4.85
**Temkin model**				
*A* (L/g)	17.81	2.05	1.95	24.78
*b* (J/mol)	77.67	70.61	155.82	133.92
*R*^2^	0.921	0.896	0.984	0.952
ARE (%)	4.01	10.91	1.48	2.14

Moreover, the maximum arsenic adsorption capacities on different iron hydroxides were compared as summarized in Table 4. The excellent arsenic removal performance of As(V) and As(III) were superior than those of other Fe-based adsorbents, which could mainly be attributed to the benefits of the petaline-like nanostructures. It should be noted that the adsorption capacities of As(V) and As(III) by the iron hydroxide nanopetalines were greater than those of conventional ferrihydrite^21,29^. These results indicated the specific potential application of arsenic removal from As-containing water.

**Table 4:** Comparison of the maximum As(V) and As(III) adsorption capacities of various iron hydroxides.

Adsorbent	pH	*q*_max_ (mg/g)		Ref
		As(V)	As(III)	
Ferrihydrite	3.0	142.86	n.a.	^21^
	6.0	71.43	n.a.	
Mg-Fe-Ala-LDH	6.0	49.8	23.6	^46^
Fe–Mn composite	5.0	69.75	132.75	^47^
CF@Mn-FeOOH	7.0	107.3	152.5	^10^
Fe–Mn composite oxide	7.0	31.68	59.44	^48^
α-FeOOH QDs@GO	n.a.	42.54	147.38	^49^
β-FeOOH NRs/CF monolith	6.0	172.9	103.4	^25^
Ca-Al-Fe ternary composites	n.a.	n.a.	56.86	^50^
Starch functionalized maghemite	n.p.	n.a.	8.6	^35^
S–nZVI	7	89.29	79.37	^51^
Fe-modified biochar	n.a.	48.57	121.61	^52^
Iron oxide coated hollow poly(methylmethacrylate)	n.a.	n.a.	10.031	^36^
AlZn-BC	n.a.	11.786	10.728	^37^
Iron hydroxide nanopetalines	4.0	217.76	91.74	Present study
	8.0	187.84	147.06	

*n.a.* not available, *n.p.* neutral pH.

#### Adsorption thermodynamics

In order to investigate the influence of temperature (303, 313 and 323 K) on As(V) and As(III) removal by the adsorbent at different initial concentrations, the thermodynamics adsorption experiments were carried out similarly to the adsorption isotherms except that the contact time was 8 h. The values of Gibbs free energy (ΔG, kJ/mol), enthalpy (ΔH, kJ/mol), and entropy (ΔS, kJ/(mol∙K)) of adsorption were evaluated by the linearized Van’t Hoff equations as follows^31,40^:

8 $$\Delta G = - RT\ln K_{c} = \Delta H - T\Delta S$$

9 $$K_{c} = \frac{{q_{e} }}{{C_{e} }}$$

10 $$\ln \frac{{q_{e} }}{{C_{e} }} = - \frac{\Delta H}{{RT}} + \frac{\Delta S}{R}$$

where notations have their usual meaning as stated above. The corresponding parameters including ΔG, ΔH and ΔS at different initial As concentrations were summarized in Table 5. The positive values of ΔH and ΔS indicated that the adsorption process was endothermic and the randomness was increased at the interface of the solid surface and arsenic species in solution. Moreover, the negative values of ΔG inferred that the adsorption process was spontaneous at all experimental temperatures. The lower values of ΔG at the higher temperature also indicated that the adsorption was favourable at the higher temperature. However, the increased value of ΔG with the increasing initial concentration at the fixed temperature could be attributed to the difficulty of availability of sorption sites at higher concentration.

**Table 5:** Thermodynamics parameters for As(V) and As(III) adsorption onto iron hydroxide nanopetalines at pH 4.0.

Concentration (mg/L)	ΔH (kJ/mol)	ΔS (kJ/(mol K))	ΔG (kJ/mol)		
			303 K	313 K	323 K
**As(V)**					
75	11.163	0.0556	− 5.700	− 6.244	− 6.813
90	11.393	0.0553	− 5.333	− 5.973	− 6.440
110	5.1121	0.0302	− 4.080	− 4.265	− 4.684
**As(III)**					
75	13.025	0.0463	− 1.097	− 1.258	− 2.023
90	12.229	0.0422	− 0.614	− 0.881	− 1.459
110	9.790	0.0329	− 0.213	− 0.411	− 0.870

#### Effect of solution pH

**Figure 5:**
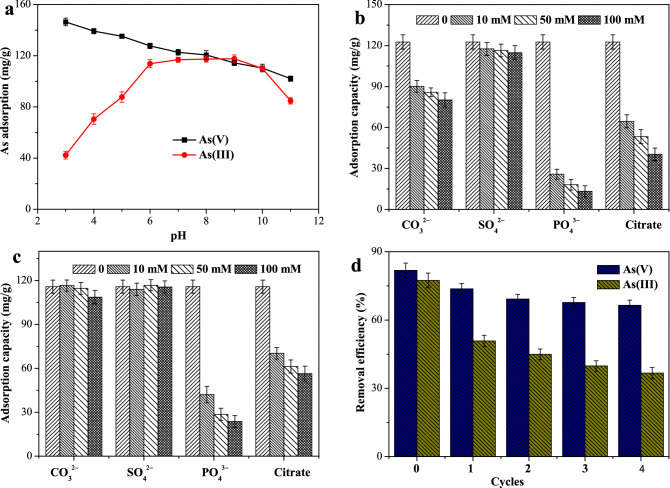
(**a**) Influence of solution pH on the removal of As(V) and As(III) with initial As concentrations at 75 mg/L and dosage of 0.5 g/L. Effect of competitive anions on As(V) (**b**) and As(III) (**c**) removal at pH 7.0 with initial As concentrations at 75 mg/L and dosage of 0.5 g/L. (**d**) Reusability of iron hydroxide nanopetalines on the removal of As(V) and As(III) with initial As concentrations at 75 mg/L and dosage of 0.5 g/L.

Solution pH can significantly affect both zeta potential of the adsorbents and arsenic species. Consequently, the removal efficiencies of the adsorbents evidently depend on the solution pH. The influence of solution pH on the uptake of As(V) and As(III) was evaluated at different pH values in the range of 3‒11 with initial As concentrations at 75 mg/L. As depicted in Fig. 5a, it is clear that the uptake of As(V) and As(III) was remarkably related to the solution pH. In the pH range of the experiments, the adsorption capacities for As(V) were comparatively higher than those for As(III), except for those at pH ~ 9.0. Similar results have been reported for other iron hydroxides, such as CF@FeOOH^10^. The reason could be attributed to the stronger affinity of As(V) for the adsorption sites on the surface of iron hydroxides than that of As(III)^20,28^. The adsorption capacity of As(V) decreased dramatically with increasing pH. The greatest removal efficiency of As(V) was observed at pH 3.0, and approximately 97.5% of the total As concentration was removed. Even though the solution pH was up to 11.0, the removal efficiency could yet reach 68%. Different from As(V) adsorption, the amount of As(III) removal increased from 3 to 6 and a broad sorption maximum was observed at pH 6‒9, after which the adsorption reduced with further increases in pH. This observation was in agreement with previous reports of ferrihydrite in the literatures^20,28^. The trends of As(V) and As(III) removal could be attributed to the surface charge of the material and arsenic species in solution, which significantly depended on the solution pH^42,47^. In the pH range of the experiments (3‒11), H_2_AsO_4_^‒^ and HAsO_4_^2‒^ were the predominant species in solution. The hydroxyl groups on the surface of the adsorbent were protonated and positively charged at pH < pH_pzc_. Thus, electrostatic attraction between As(V) and adsorption sites enlarged at lower pH values, and therefore the removal efficiencies increased. However, at pH > pH_pzc_, the deprotonation of the surface gradually dominated, causing the negative surface charge. This led to the enhancement of electrostatic repulsion, which would be harmful to arsenic removal. Herein, the uptake of As(V) reduced with the increases of solution pH. Different from As(V) species in solution, neutral H_3_AsO_3_ was the dominant form at pH < 9.2 and H_2_AsO_3_^‒^ were the main species at pH 9–11 according to the p*K*_a1_ (~ 9.2) of As(III)^2^. With increasing pH from 3 to 9, the electrostatic attraction between As(III) and adsorption sites became stronger, the adsorption capacities increased, and a broad sorption maximum could be found at pH 6‒9. With further increases of pH in solution, the stronger electrostatic repulsion resulted into the sharp decreases in As(III) removal. Similar trends of As(V) and As(III) removal have been reported in previous literatures, such as Fe–Zr binary oxide^17^, and ferrihydrite^20^.

#### Effect of co-existing oxyanions

Anions, such as bicarbonate, sulfate, phosphate, and natural organic acids, generally coexist with arsenic in natural or industrial water systems, which could interfere in the arsenic removal for competitive adsorption. In this study, the influence of CO_3_^2–^, SO_4_^2–^, PO_4_^3–^ and citrate, as a representative of natural organic acids, on the arsenic removal were investigated at pH 7.0 with initial phosphate concentrations of 75.0 mg/L. Figure 5b,c present the experimental results. Obviously, it can be seen that the co-existing SO_4_^2–^ at different concentrations exhibited a slight effect on adsorption capacity of As(V) (Fig. 5b). Only less than 6% of As(V) removal efficiency was reduced even when the concentration of sulfate was as high as 100 mM. Whereas, CO_3_^2–^, PO_4_^3–^ and citrate showed obvious inhibitory effects on As(V) removal. The presence of CO_3_^2–^ decreased the adsorption capacity of As(V) more remarkably than SO_4_^2–^. The adsorption capacity of As(V) significantly decreased from 122.6 to 80.2 mg/g as the concentration of CO_3_^2–^ was increased from 0 to 100 mM. Specifically, the co-existing PO_4_^3–^ and citrate manifested significant influences on the uptake of As(V) by the material, and PO_4_^3–^exhibited the greatest impact.

As described in Fig. 5c, compared to that of As(V), the adsorption of As(III) was slightly affected by these anions, which could be attributed to the different affinities of arsenic species for mineral surface sites^20,28^. There was a negligible impact by the presence of SO_4_^2–^ and little interference from CO_3_^2–^. In general, the inhibiting influences of these anions on As(III) adsorption followed the order of PO_4_^3–^ > citrate > CO_3_^2–^ > SO_4_^2–^, which was the same as those on As(V) adsorption. The most significant reduction of arsenic uptake in the presence of phosphate has also been observed in previous researches^10,12,28,40^. This was attributed to the effective competition between arsenic species and phosphate due to their similarities in coordination geometry and geochemical behaviour^28^.

#### Regeneration and reusability

In order to estimate the applicability of iron hydroxide nanopetalines from an economical perspective, the reusability of the material was investigated by four consecutive adsorption-regeneration experiments at pH 7.0. 2.0 M NaOH solution was used as the regenerant in the regeneration experiments. As depicted in Fig. 5d, the removal efficiencies of As(V) and As(III) after the first cycle were about 73.7% and 50.8%, compared to about 81.4% and 77.4% of those of virgin adsorbent, respectively. After four cycles, more than 67% of removal efficiency of As(V) was maintained, whereas the removal efficiency of As(III) was dramatically reduced to 37%. This significant discrepancy could be attributed to the stronger affinity of As(V) for the mineral surface sites than that of As(III)^28^. These results indicated that this material was promising applications in treatment of As(V)-contaminated water, whereas much more work is expected to treat As(III)-containing water in the regeneration and reusability process, such as, the preoxidation of As(III).

### Analyses of the adsorption mechanism

Based on the above batch experimental results of As(V) and As(III) removal, FTIR, p-XRD and XPS, were performed to characterize the iron hydroxide nanopetalines before and after arsenic removal in order to understand the adsorption mechanism of As(V) and As(III).

**Figure 6:**
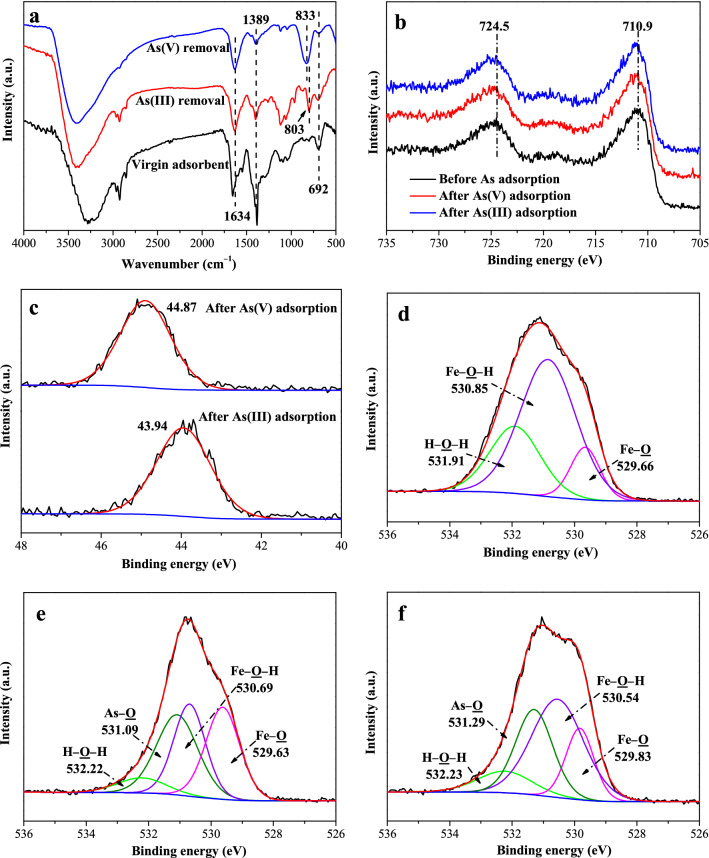
(**a**) FTIR spectra of the iron hydroxide nanopetalines before and after As(III) and As(V) adsorption at pH 4.0. (**b**–**f**) XPS analyses of the iron hydroxide nanopetalines before and after arsenic adsorption at pH 4.0. Fe 2p (**b**), As 3d (**c**), O 1s (**d**, before As adsorption; **e**, As(V) adsorption; **f**, As(III) adsorption) high-resolution XPS spectra before and after As(V) and As(III) removal.

FTIR spectra of the iron hydroxide nanopetalines before and after As(V) and As(III) removal are described in Fig. 6a. Generally, the peaks located at 3600‒3200 cm^−1^ are ascribed to the stretching vibration of the ‒OH groups, and the bands at around 1634 cm^−1^ are assigned to the deformation vibration of physisorbed water molecules^10,39,42,53^. These observations suggested that physisorbed water was present in all samples. Peaks centered at 1388 cm^−1^ could be attributed to the vibration of residual NO_3_^−^ anions for the use of the raw synthesis materials of Fe(NO_3_)_3_∙9H_2_O^19^. The bands observed at around 1125 and 692 cm^−1^ are characteristics of the hydroxyl groups bonding with Fe atoms (Fe–OH bonds)^10,39^. After the uptake of As(V) and As(III), new peaks at 833 and 803 cm^−1^ for As(V) and As(III) appeared, respectively, which could correspond to the As–O stretching vibration^38,39^. It is clearly observed that the intensities of peaks at approximately 1125 and 692 cm^−1^ significantly decreased. This indicated that the amount of the hydroxyl groups (Fe–OH bonds) after arsenic adsorption reduced and that chemical interactions between arsenic and the iron hydroxide nanopetalines happened, resulting in the formation of inner-sphere surface complexes^28,39^. Therefore, the ligand exchange reactions of hydroxyl groups between arsenic and Fe–OH groups were a crucial mechanism during the arsenic elimination process. Furthermore, the intensities of peaks at about 1125 and 692 cm^−1^ for As(III) were stronger than those for As(V), which suggested that the adsorption capacity of As(V) was greater than that of As(III) at pH 4.0. This observation was further confirmed by the batch experiments mentioned above.

As shown in Fig. S1, the XRD spectra of the iron hydroxide nanopetalines before and after arsenic adsorption at pH 4.0 and 8.0 exhibited no obvious changes, which indicated that the material was stable in the process of arsenic removal. Previous studies demonstrated that amorphous ferric arsenate precipitated on the surface of ferrihydrite after arsenate adsorption at acidic pH values (3, 4)^21,39^. The lack of an appreciable effect after arsenic adsorption in our research in the XRD spectra could be due to the weak crystallinity of ferric arsenate precipitate on the surface.

As displayed in Figs. S2 and 6b‒f, X-ray photoelectron spectroscopy was performed to gain further insight into the surface electronic structure and bonding configuration of the samples. XPS data including full, Fe 2p, As 3d, and O 1s high-resolution spectra for the original adsorbent before and after As(V) and As(III) adsorption at pH 4.0 were investigated. Fig. S2 illustrates the survey XPS spectra, exhibiting the presence of Fe, O and C elements. With the material adsorbing As, new peaks with a binding energy at ~ 44.5 eV appeared, proving the successful adsorption of arsenic by the iron hydroxide nanopetalines. The XPS spectra of Fe 2p (Fig. 6b) show the characteristic peak positions of Fe at 710.9 eV for Fe 2p1/2 and 724.5 eV for Fe 2p2/3, respectively, which were in accordance with previous reports^10,54^. The binding energies of Fe 2p shifted to higher energies after As(V) and As(III) adsorption, which could be attributed to the formation of Fe‒O‒As bonds during the uptake of arsenic^10,25^. The high resolution As 3d spectra of the iron hydroxide nanopetalines after As(V) and As(III) adsorption are described in Fig. 6c. Two peaks at 44.87 eV and 43.94 eV were observed, which were the characteristic peaks of As(V) and As(III), respectively^12,55^.

Figure 6d‒f depict the high resolution scans of O 1s spectra before and after As(V) and As(III) adsorption, whose peaks were obtained at 531.06 eV, 530.67 eV and 530.74 eV. The peak positions in the O 1s spectra shifted to the lower binding energies after arsenic adsorption, which might be due to the formation of Fe‒O‒As complexation^10,25^. As shown in Fig. 6d, the high resolution scan of O1s spectrum of the virgin adsorbent could be decomposed into three peaks at 529.66 eV, 530.85 eV and 531.9 eV, which could correspond to different oxidation forms of metal oxides (Fe–O), hydroxyl groups bonded to the metal (Fe–OH) and the adsorbed water (H_2_O), respectively^12,42,49,55^. After As(V) and As(III) removal (Fig. 6e,f), new component peaks could be found at 531.09 eV and 531.29 eV, respectively, which can be identified as arsenic-oxygen bonds (As‒O)^10,12,55^. The relative area ratios of the peaks assigned to As–O bond were 32.9% and 27.7% after the adsorption of As(V) and As(III), respectively, in line with the fact that the amount of As(V) adsorption was much higher than that of As(III) adsorption at pH 4.0. What’s more, the relative content of Fe–OH groups remarkably decreased from 62.8% for the original sample to 28.9% and 44.6% for the samples after As(V) and As(III) removal, respectively. These results suggested that As(V) and As(III) were removed by the iron hydroxide nanopetalines through surface hydroxyl exchange reactions of the Fe–OH groups with arsenic species, and then, the inner-sphere surface complexes were formed, which further confirmed the removal mechanism of the surface hydroxyl exchange reactions observed by the abovementioned FTIR study (Fig. 4a). This observation has also been demonstrated in previous reports on phosphate and/or arsenic removal^25,41,42,54,56^.

Based on the above discussion from the batch experiments and FTIR, XRD and XPS analyses, the ligand exchange reactions between the hydroxyl groups on the surface of the iron hydroxide nanopetalines (Fe–OH) and arsenic species at pH 4.0 and 8.0 were likely the predominant removal mechanism of arsenic described as follows (where ≡S represents the surface):

At pH = 4.0, H_2_AsO_4_^‒^ was the predominant species in solution for As(V), and neutral H_3_AsO_3_ was the predominant form for As(III):

11 $$2[ \equiv {\text{S}}{- }{\text{OH}}_{{2}}^{ + } {\text{] (s) }} + {\text{ (HO)}}_{{2}} {\text{AsO}}_{{2}}^{ - } \, \to \, ( \equiv {\text{S}}{-}{\text{O}} - )_{2} {\text{As}}{-} ({\text{OH}})_{2} + {\text{H}}_{2} {\text{O}}$$

12 $$2[ \equiv {\text{S}} {-} {\text{OH}}_{{2}}^{ + } {\text{] (s) }} + {\text{ (HO)}}_{{2}} {\text{AsO }} \to \, ( \equiv {\text{S}} {-} {\text{O}} - )_{2} {\text{As}} {-} {\text{OH}} + {\text{H}}_{2} {\text{O}}$$

At pH = 8.0, the main species of As(V) and As(III) were HAsO_4_^2‒^ and H_3_AsO_3_, respectively:

13 $$2[ \equiv {\text{S}} {-} {\text{OH}}_{{2}}^{ + }] {\text{ (s) }} + {\text{ HOAsO}}_{{3}}^{2 - } \, \to \, ( \equiv {\text{S}} {-} {\text{O}} - )_{2} {\text{As}} {-} {\text{O}}_{2}^{2 - } + {\text{H}}_{2} {\text{O}}$$

14 $$2[ \equiv {\text{S}} {-} {\text{OH}}_{{2}}^{ + }] {\text{ (s) }} + {\text{ (HO)}}_{{2}} {\text{AsO }} \to \, ( \equiv {\text{S}} {-} {\text{O}} - )_{2} {\text{As}} {-} {\text{O}}^{ - } + {\text{H}}_{2} {\text{O}}$$

## Conclusion

In general, iron hydroxide nanopetalines were developed by a precipitation process using KBH_4_ and applied to the treatment of As(V) and As(III) contaminated water. The adsorption batch experiments indicated that the performance of iron hydroxide nanopetalines in arsenic removal was more excellent than that of ferrihydrite. The adsorption rates were superior rapid in the first 6 h and gradually slowed down and could reach equilibria after 24 h. At pH values of 4.0 and 8.0, the maximal adsorption capacities of As(V) calculated by the Langmuir model were 217.76 and 187.84 mg/g, respectively, and those of As(III) were 91.74 and 147.06 mg/g, respectively. The solution pH and presence of PO_4_^3–^ and citrate can significantly affect the As(V) and As(III) removal efficiencies. More than 67% of the removal efficiency of As(V) could be easily maintained after four cycles, indicating that the material could be effectively considered reusable. The surface morphology and crystal structure before and after arsenic removal characterized by SEM and XRD analyses were stable. The FTIR, XRD and XPS analyses suggested that the dominant mechanism of arsenic removal was the surface hydroxyl exchange reactions of Fe–OH groups with arsenic species and the inner-sphere surface complexes were formed. The highly efficient performance in scavenging arsenic indicates the great potential for application in the treatment of As-containing water, such as As-containing industrial wastewater. The developed material also has a possible application in the remediation of arsenic contaminated soil due to the high affinity and high capacities for arsenic, which needs further research.

## Supplementary Information

Supplementary Figures.

## Data Availability

The datasets supporting the conclusions of this article are included within the article.
