# Supplementary material for: Arsenic removal performance and mechanism from water on iron hydroxide nanopetalines
Source: Sci Rep. 2022 Oct 14;12:17264. doi: 10.1038/s41598-022-21707-1 (PMC9568553; doi:10.1038/s41598-022-21707-1)
Supplement: Supplementary file 1 — Supplementary Figures. [file 41598_2022_21707_MOESM1_ESM.docx]

**Supporting Information**

**Arsenic removal performance and mechanism from water on iron hydroxide nanopetalines**

Yulong Wang^1,2,3,†^, Lin Zhang^1,3,†^, Chen Guo^1,3^, Yali Gao^1,3^, Shanshan Pan^1,3^, Yanhong Liu^4,^*, Xuhui Li^1,2,3,^*, Yangyang Wang^1,2,3,^*

^1^ National Demonstration Center for Environmental and Planning, College of Geography and Environmental Science, Henan University, Kaifeng, 475004, China

^2^ Key Laboratory of Geospatial Technology for the Middle and Lower Yellow River Regions (Henan University), Ministry of Education, Kaifeng, 475004, China

^3^ Henan Engineering Research Center for Control and Remediation of Soil Heavy Metal Pollution, Henan University, Kaifeng, 475004, China

^4^ College of Software, Henan University, Kaifeng, 475004, China

^†^ These two authors contribute equally to this work and should be regarded as co-first authors.

*Corresponding authors: [liuyanhongxmu@163.com](mailto:liuyanhongxmu@163.com) (Y Liu); [lixuhui@henu.edu.cn](mailto:lixuhui@henu.edu.cn) (X Li); [wangyangyangxyz@163.com](mailto:wangyangyangxyz@163.com) (YY Wang).

**Figure S1.** XRD patterns of iron hydroxide nanopetalines before and after As(III) and As(V) adsorption at pH 4.0 and 8.0.

**Figure S2.** Full spectra of iron hydroxide nanopetalines before and after As(III) and As(V) adsorption at pH 4.0.
